# Editorial for the Special Issue Recognition Robotics

**DOI:** 10.3390/s23208515

**Published:** 2023-10-17

**Authors:** José María Martínez-Otzeta

**Affiliations:** Department of Computer Science and Artificial Intelligence, University of the Basque Country, 20018 Donostia-San Sebastián, Spain; josemaria.martinezo@ehu.eus

Perception of the environment is an essential skill for robotic applications that interact with their surroundings. Alongside perception often comes the ability to recognize objects, people, or dynamic situations. This skill is of paramount importance in many use cases, from industrial to social robotics. Robots that can accurately perceive and understand their environment are critical for tasks like manufacturing, delivery, healthcare, and assisting humans in homes or public spaces. Object recognition enables robots to identify items, tools, and obstacles in their vicinity. This allows industrial robots to select the right parts or manipulators, logistics robots to handle packages, and autonomous vehicles to avoid collisions. Activity recognition allows robots to interpret human motions and behavior. This facilitates safe and intuitive collaboration in shared workspaces. It also permits service robots to determine user intents and respond appropriately. Person recognition provides robots the means to identify individuals. This capability supports applications like personalized assistance, healthcare monitoring, and security surveillance. Altogether, these skills comprise the fundamental building blocks for robots to operate adaptively in the real world.

This Special Issue “Recognition Robotics” of *Sensors* seeks to explore new research proposals on this increasingly important topic. The fifteen accepted papers in this issue cover human–robot collaboration [1], person re-identification [2,3], human–robot interactions [4,5], visual servoing [6], cooperative mapping [7], semantic segmentation [8,9], object classification [10], multi-object tracking [11], robot path planning [12], embedded deep learning [13], activity recognition [14], and robust model fitting [15]. These works present novel techniques using tools such as fuzzy logic, deep learning, computer vision, ultrasonic sensing, spline optimization, and more to advance robot capabilities in real-world conditions. The research aims to overcome challenges in uncertainty, limited data, computational constraints, and complexity across various application domains. In summary, this Special Issue provides a sampling of the latest innovations and progress in enabling robots that can effectively perceive, learn, plan, manipulate, and collaborate in unstructured environments through advances in recognition capabilities.

In [1], Yalçinkaya et al. introduce a Fuzzy State-Long Short-Term Memory (FS-LSTM) approach for human–robot collaboration in dynamic fields like agriculture and construction. These tasks are time-consuming and risky for humans, making robotic assistance valuable. The method handles the ambiguity in human behavior by fuzzifying sensory data and employing a combined activity recognition system using state machines and LSTM. Experimental validation showed that FS-LSTM outperforms traditional LSTM in accuracy and computational efficiency.

In [2], Casao et al. introduce an unsupervised method for person re-identification, capable of automatically adding new identities to an adaptive gallery in open-world settings. The system compares current models to new unlabeled data and uses information theory to keep compact representative models. Experimental results, including comparisons to other unsupervised and semi-supervised methods, validate the effectiveness of their approach.

The authors of [3] propose a lightweight deep metric learning technique for reliable person re-identification, aimed at robot tracking. This method addresses challenges like clothing and pose changes by employing a novel attention mechanism. This focuses on specific body parts, retains global context, and enables cross-representations for robust identification. The experimental results show up to 80.73% and 64.44% top-rank accuracy, outperforming existing methods. The authors suggest that integrating this metric improves tracking reliability in dynamic environments.

In [4], Błażejowska et al. explore the impact of emotional feedback from the Miro-E robot on high school students during a programming education session. The robot monitored students’ emotions via facial expression analysis and provided affective feedback like verbal praise and tail wagging. Compared to a control group with neutral robot responses, the emotional feedback positively impacted engagement, particularly for students with little prior programming experience. However, it also slightly reduced the robot’s likeability, hinting at an uncanny valley effect. Due to a small sample size, the study focused on qualitative insights.

In [5], Marques-Villarroya et al. introduce a robotic perception architecture that employs bio-inspired endogenous attention to improve human–robot interactions. The architecture uses multisensory inputs and ranks stimuli based on their relevance to the robot’s tasks, particularly emphasizing human presence and actions. By doing so, it optimizes the robot’s focus and behavior, leading to more efficient interactions. Implemented on the Robot Operating System (ROS), the architecture demonstrates strong real-time performance and extensibility. The authors argue that this bio-inspired approach enhances the robot’s responsiveness while reducing complexity.

In [6], Marchionna et al. demonstrate how a low-cost, six-axis robotic arm, e.Do, can play Jenga using instance segmentation and visual servoing. The system employs an affordable RGB-D camera and force sensor. A customized deep learning model is trained to identify each Jenga block, enabling precise visual tracking during manipulation. The force sensor helps decide if a block can be safely removed. Testing shows up to 14 consecutive successful block extractions before the tower collapses. The authors note that Jenga serves as a complex benchmark, driving advancements in multi-step reasoning, integrated sensory perception, and high-precision control.

The authors of [7] propose a decentralized framework for collaborative 3D mapping using mobile robots with LiDAR sensors in large-scale outdoor settings such as agriculture and disaster response. The real-time method allows robots to share and merge locally scanned submaps into a global map, even with limited communication bandwidth. A conditional peer-to-peer strategy is used for sharing map data over different distances. Experiments in a real-world solar power plant confirm the approach’s efficiency and reliability for multi-robot mapping of extensive outdoor areas.

In [8], Pinkovich et al. address the challenge of autonomously selecting safe landing sites for delivery drones in dense urban areas. Their multi-resolution technique captures visual data at varying altitudes, enabling both wide exploration and high-resolution sensing. A semantic segmentation deep neural network processes this data, updating probability distributions for each ground patch’s landing suitability. When a location’s confidence exceeds a threshold, it is selected as viable. The authors find the method effectively balances the trade-off between exploration and resolution in constrained urban environments.

Lee et al. introduce in [9] an “Extract-Append” data augmentation technique to boost the accuracy of models detecting wild animals in agricultural fields. The method uses semantic segmentation to isolate animal shapes from sample images and combines them with new backgrounds to enrich the training dataset. Testing shows at least a 2.2% improvement in mean Average Precision over traditional methods, and the technique enables ongoing flexible data augmentation.

The authors of [10] present vision-based methods for automated recycling of used electronic components such as capacitors and voltage regulators. Using a custom object detection algorithm, they identify key areas in cluttered workspaces and compare three classification techniques: SNNs, SVMs, and CNNs. After hyperparameter tuning, CNNs prove to be the most accurate with a 98.1% success rate, making them the preferred method for reliable automated recycling.

Paper [11] proposes the use of deep neural networks to detect illegal garbage dumping in urban areas. They combine OpenPose for human pose estimation, YOLO for garbage bag classification, and DeepSORT for object tracking. The system measures the distance between a person’s wrist and the garbage bag to determine illegal dumping. Experimental results show their method offers higher accuracy and lower false alarms compared to other approaches, making it effective for automated monitoring against unlawful waste disposal.

In the research presented by Rykała et al. [12], a path-planning method is developed for an unmanned ground vehicle (UGV) to follow a human guide using ultra-wideband (UWB) technology. They use smoothing splines to reconstruct the guide’s path from periodic distance measurements. The approach is computationally efficient and can handle missing data, making it suitable for real-time applications.

The authors of [13] provide a comprehensive evaluation of how well state-of-the-art deep learning object detection models perform on embedded electronics. They assess multiple architectures and quantization techniques to make the models more efficient for embedded and robotics applications. The paper outlines the entire process from model conversion to deployment and performance measurement on embedded devices. It offers guidelines for choosing the right hardware and optimization strategies, and discusses the various factors that influence performance in real-time robotics systems.

In [14], Strazdas et al. introduce RoSA, a framework that facilitates human–robot interactions using speech and gestures. Running on ROS, the system incorporates speech recognition, face identification, and pose estimation. A user study revealed that RoSA’s usability was on par with a human-controlled setup, suggesting it offers a natural interaction experience. The authors highlight the value of multi-sensory integration for more human-like and flexible robot interactions.

The authors of [15] review the RANSAC algorithm’s applications in robotics, focusing on shape detection and feature matching. They explore various enhancements to RANSAC that improve its speed, accuracy, and robustness. The survey also discusses trade-offs between computational cost and performance, highlights recent robotics applications, and provides a list of open-source RANSAC libraries. The survey offers robotics researchers and developers an extensive reference on the state of the art in RANSAC techniques.

In summary, the fifteen papers in this Special Issue on “Recognition Robotics” demonstrate the tremendous progress being made in enabling robots to effectively perceive, understand, plan, and interact in the real world. However, significant challenges remain before these innovative techniques can be reliably deployed in unconstrained environments. Testing novel algorithms in controlled simulations or lab settings with simplified assumptions can be deceptively promising, because applying recognition capabilities on physical robotic platforms in complex dynamic scenarios reveals many subtleties. Interactive testing is critical to expose limitations around uncertainty, variability, and computational constraints. Moving innovations out of the lab or controlled scenarios requires addressing edge cases and graceful failure modes, and therefore there is still substantial effort needed to robustly handle the diversity and unpredictability of the real world. Nevertheless, the field continues steadily on an exciting path towards enabling robot assistants and coworkers that can perceive, learn, reason, and collaborate at a human level. These capabilities will lead to transformative applications, and the works presented in this Special Issue provide an inspiring snapshot of the road ahead.
